# Calibration of PurpleAir PA-I and PA-II Monitors Using Daily Mean PM_2.5_ Concentrations Measured in California, Washington, and Oregon from 2017 to 2021

**DOI:** 10.3390/s22134741

**Published:** 2022-06-23

**Authors:** Lance Wallace, Tongke Zhao, Neil E. Klepeis

**Affiliations:** 1 Independent Researcher, Santa Rosa, CA 95049, USA; 2 Independent Researcher, Milpitas, CA 95035, USA; tongke1221@gmail.com; 3 Department of American Indian Studies, San Diego State University (SDSU), San Diego, CA 92182, USA; pubs4@klepeis.net; 4 Education, Training, and Research, Inc. (ETR), Scotts Valley, CA 95066, USA

**Keywords:** sensors, low-cost particle monitors, calibration factor, PurpleAir; particles, PM_2.5_, ALT-CF3, algorithm, PMS1003, PMS5003

## Abstract

Large quantities of real-time particle data are becoming available from low-cost particle monitors. However, it is crucial to determine the quality of these measurements. The largest network of monitors in the United States is maintained by the PurpleAir company, which offers two monitors: PA-I and PA-II. PA-I monitors have a single sensor (PMS1003) and PA-II monitors employ two independent PMS5003 sensors. We determine a new calibration factor for the PA-I monitor and revise a previously published calibration algorithm for PA-II monitors (ALT-CF3). From the PurpleAir API site, we downloaded 83 million hourly average PM_2.5_ values in the PurpleAir database from Washington, Oregon, and California between 1 January 2017 and 8 September 2021. Daily outdoor PM_2.5_ means from 194 PA-II monitors were compared to daily means from 47 nearby Federal regulatory sites using gravimetric Federal Reference Methods (FRM). We find a revised calibration factor of 3.4 for the PA-II monitors. For the PA-I monitors, we determined a new calibration factor (also 3.4) by comparing 26 outdoor PA-I sites to 117 nearby outdoor PA-II sites. These results show that PurpleAir PM_2.5_ measurements can agree well with regulatory monitors when an optimum calibration factor is found.

## 1. Introduction

Recently a major technological advance has occurred with the development of low-cost particle sensors [1]. Many of these monitors operate continuously, often silently, and often data are transmitted in real time to cloud databases that can be accessed through Application Programmer Interfaces (APIs). 

One example of this development is the large-scale community of users of PurpleAir monitors (https://www2.purpleair.com/, accessed on 20 June 2022). More than 20,000 PurpleAir monitors have been monitoring both outdoor and indoor fine particles at some time since 2017. Two monitor types (PA-I and PA-II) are sold by PurpleAir. The PA-I monitor is recommended for indoor use. It contains a single sensor (PMS 1003) manufactured by Plantower (http://www.plantower.com/en/, accessed on 20 June 2022). The PurpleAir PA-II monitor is recommended for outdoor use. It contains two Plantower PMS 5003 sensors. The two sensors are independent and can be compared to determine the level of agreement. Except for sites designated as private by users, data on sites, access keys, and the latest sensor readings are publicly accessible using an API provided by the PurpleAir company (https://api.purpleair.com/, accessed on 20 June 2022), with complete real-time data available via the Mathworks Thingspeak API (https://www.mathworks.com/help/thingspeak/rest-api.html, accessed on 20 June 2022). 

Multiple studies have examined the PMS 5003 sensor, either alone or as contained in the PA-II monitor [2,3]. The 1003 and 5003 sensors have been compared in several studies [4,5,6]. These studies have raised questions about how accurately PurpleAir (Plantower) sensors respond to particles of known size; for example, one study found that particles below the lower cutoff diameter of 0.3 µm created a response in the sensor [7]. Laboratory studies of multiple common indoor sources of fine particles found widely varying responses of all low-cost sensors measured, including the Plantower sensors [8,9]. The AQ-SPEC program (http://www.aqmd.gov/aq-spec, accessed on 20 June 2022) found that the PA-II monitor has good precision but PM_2.5_ readings read high by approximately 40%. One California study applied geographic regression to compare 54 PurpleAir outdoor sites with 26 regulatory monitors providing 128,777 paired PM_2.5_ measurements [10]. This study also found an overestimation of PM_2.5_ concentrations using the CF1 algorithm provided by Plantower. A USEPA study of several US locations derived a model for estimated PM_2.5_ including the effect of relative humidity; the model multiplies the CF1 estimate by about 0.54 [11]. Another study found that the CF1 algorithm required a calibration factor of 0.53, indicating a nearly 100% overestimation of PM_2.5_ concentrations [12].

Two recent studies have developed and applied an improved algorithm for calculating PM_2.5_ concentrations from PA-II monitors [13,14]. The algorithm is called ALT-CF3. This approach uses the particle numbers reported by Plantower in the three size categories 0.3–0.5 µm, 0.5–1 µm, and 1–2.5 µm. Selecting an average particle size in each category and calculating the particle volume, followed by multiplying by an assumed density (in this case, the density of water), provides an alternative estimate of PM_2.5_. This estimate for 33 outdoor PurpleAir monitors was then compared to 27 nearby regulatory monitors over a 20-month period from November 2018 to 30 June 2020 to provide a final calibration factor [13]. Four different approaches to determining the CF resulted in estimates ranging from 2.93 to 3.14, with a final estimate of 3.05 (SE 0.05).

Applying this CF to all data collected in a study of PurpleAir measurements in California, quite high R^2^ values (median 0.92 within a narrow IQR of 0.89 to 0.94) were found for 91 pairs of PA-II and regulatory monitors over typical periods of hundreds of days [14].

The results from these two studies provide evidence that even though the bias may be large, the dependability and repeatability of the response are high. Therefore, we hypothesize that a simple linear correction to the bias (the CF estimate) can produce a good agreement with research-grade instruments.

The precision of the PA-II monitors, i.e., inter-unit agreement, is a crucial input in judging their utility. Precision is defined here as the absolute difference between the A and B sensors in a single PA-II monitor divided by their sum: precision = abs(A − B)/(A + B). For two sensors, this corresponds to the relative standard deviation (RSD)/√2. The mean precision of 350,000 indoor and outdoor measured PM_2.5_ concentrations from PA-II monitors was 6%, with more than 90% of values showing a precision better than 20% [13]. The median or geometric mean precision was 4–5%. The independence of the two PMS 5003 sensors makes it possible to identify observations with poor precision. This allows investigators to filter data by selecting a cutoff precision.

The ALT-CF3 algorithm performs better than the Plantower CF_1 algorithm (smaller bias, better precision, higher accuracy, lower limit of detection, improved distribution characteristics) [13]. The ALT-CF3 algorithm has been made available for download as an alternative to the Plantower algorithms on the PurpleAir API site. On this API site, the ALT-CF3 algorithm is given the name PM_2.5__alt. This option was not available at the time we downloaded all data; we calculated hourly average PM_2.5_ estimates using the ALT-CF3 algorithm. Of various alternatives offered by PurpleAir, the ALT-CF3 algorithm is the only one that does not incorporate the proprietary Plantower algorithms described in [15].

In this paper, we determine a revised calibration factor for PA-II monitors using all PurpleAir monitors located near regulatory PM_2.5_ Air Quality System (AQS) monitors using FRM in the West Coast states of Washington, Oregon, and California between the dates of 1 January 2017 through 8 September 2021. We also determine a new calibration factor for PA-I outdoor monitors by comparing them to nearby outdoor PA-II monitors.

## 2. Materials and Methods

### 2.1. Obtaining the Data

We created a custom R package containing scripts to automate downloads of hourly data using the API for the Mathworks ThingSpeak cloud database for PurpleAir data and compute hourly PM_2.5_ concentrations from size-specific particle counts. The R package was also used to compute daily mean PM_2.5_ concentrations from the downloaded hourly data, using the ALT-CF3 algorithm. We required at least 18 valid hourly measurements for a given daily mean to be accepted into the final database. We removed negative numbers, zeros, “NA” notations, and duplicates. We also removed readings for the PA-II indoor and outdoor measurements if the two independent “A” and “B” PMS-5003 sensors did not agree within a precision of 20%, matching the limit chosen in [13,14]. Also, individual sites were required to have at least 30 daily PM_2.5_ averages. Our present dataset runs over a 56-month period from January 2017 to 8 September 2021 in three U.S. States—Oregon, Washington, and California. We used the ALT-CF3 algorithm, as described above, to adjust PM_2.5_ for all downloaded data.

There is a direct relationship between the number of particles per deciliter in the three smallest size categories (N1, N2, N3) and the ALT-CF3 PM_2.5_ estimate. This relationship is

PM_2.5_ = 3(0.00030418 × N1 + 0.0018512 × N2 + 0.02069706 × N3) (1)

The number of particles per deciliter N1 in the smallest size category is found by subtracting the Plantower-provided number “≥0.5 − 1 µm” from the number “≥0.3 − 0.5 µm”. N2 and N3 are found by similar subtractions from the next-higher sets of size categories. The coefficients shown derive directly from the ALT-CF3 approach of selecting an average (geometric mean) diameter for the three smallest size categories and using the density of water. The factor of 3 shown is the CF; any change in the CF would only affect that factor and not the other coefficients.

No computation is required to download the PM2.5_alt variable in the PurpleAir API site; PurpleAir staff have already performed the operation using the equivalent of Equation (1). Also, the “ALT-CF3” algorithm, available as an alternative “conversion factor” on the PurpleAir mapping page has also had the above computation performed by the PurpleAir staff.

### 2.2. Federal and State Agency Data

AQS sites operate two main types of monitors. The FRM collects a single sample over 24 h on a filter that is weighed under strict conditions of relative humidity (RH) and temperature. The Federal Equivalent Method (FEM) measures hourly PM_2.5_ concentrations. Daily average FRM/FEM PM_2.5_ concentrations are accessible at https://aqs.epa.gov/aqsweb/airdata/download_files.html (accessed on 20 June 2022).

For the years 2017 through 2020, the FRM and FEM data were downloaded from that site. For the year 2021 (up to 8 September), the data were obtained from various State and Regional Agencies. These State and Regional Agencies apply local Quality Assurance/Quality Control (QA/QC) requirements, which may differ somewhat from the national QA/QC methods. Therefore, the 2021 data supplied to us may include some values that have later been changed as a result of QA/QC alterations.

### 2.3. Recalibration of PA-II Monitors with PMS-5003 Sensors

We re-examined the estimated calibration factor of 3.0 for PA-II monitors using a larger sample of PurpleAir monitors over a longer period (>4 years) than previously used [13].

Our general approach to determining the revised calibration factor for outdoor PA-II monitors is outlined here:

1. Identify all PurpleAir sites within a specified distance of the target site (e.g., an FRM site). We use several possible distances (0.5, 1, 2, 10 km) to see how distance affects correlations.
2. Download the hourly average PM_2.5_ data calculated using the published calibration factor of 3 in the ALT-CF3 or “PM2.5 alt” algorithms
3. Regress the PM_2.5_ daily measurements at these sites on the target (regulatory) site PM_2.5_ measurements
4. Find the best-fitting (revised) calibration factor by minimizing the mean absolute error (MAE) or the root mean squared error (RMSE) for all pairs of sites. We include both measures to estimate their different effects on the estimated CF.

### 2.4. New Calibration of PA-I Monitor with PMS-1003 Sensor

Because the PurpleAir company offers its PA-I monitor specifically for use indoors, there are many more PA-I monitors used indoors than PA-II monitors (3191 compared to 981) over our 4.7-year period. To our knowledge, the PA-I monitors do not have a recognized calibration factor. Therefore, we developed a CF specifically for this sensor. Since indoor sites have no expected similarity to nearby indoor sites, we were limited to comparing ***outdoor*** PA-I sites to nearby outdoor PA-II sites.

For this case, there are too few PA-I outdoor sites to be able to compare with nearby regulatory sites. Instead, we compare with nearby PA-II outdoor sites using step 3 above, except that the “target” site is now a PA-II outdoor site. We required (1) at least 30 days of joint measurements and monitor operation greater than or equal to 18 h each day and (2) that the two sensors in the PA-II monitors have a precision better than 20%. Step 4 above provides a calibration factor for the PA-I monitors *that is related to the calibration factor* of the PA-II monitors. 

## 3. Results

### 3.1. Recalibration of PA-II Monitors by Comparison with Regulatory Monitors

Following the quality control measures described above, the PurpleAir PA-II outdoor data consisted of 83,304,477 million hourly observations at 10,235 sites. Requiring the precision to be better than 20% reduced the total number to 77,277,831 hourly observations at 9347 sites, a loss of 7.2% of all observations. These were averaged to provide 3,529,229 daily observations (Figure S1 in the Supplementary Information). Daily mean measurements from 95 Federal and State regulatory monitors in the three-state region were also obtained (Figure S2). The outdoor mean PM_2.5_ concentrations and related statistics from these two fundamental datasets are provided (Tables S1 and S2).

From these PA-II sites, we selected all those within a distance of 0.5 km from a regulatory monitor to match previous work [13]. 182 PA-II outdoor monitors and 47 regulatory monitors in three states provided 39,494 daily PM_2.5_ averages for pairs of sites averaging about 300 days of joint measurements. We regressed the PurpleAir PA-II estimates of PM_2.5_ on those of the regulatory monitors, using the published value of 3.0 as the calibration factor for the ALT-CF3 algorithm. The minimum root mean squared error (RMSE) of 2.14 µg/m^3^ occurred at a CF of 1.11 times the old CF of 3 and the minimum mean absolute error (MAE) of 1.46 µg/m^3^ occurred at a CF of 1.13 times the old CF. These values would support an upward revision of 11–13% applied to our previous CF of 3.0, or a new estimated CF in the range of 3.33 to 3.39, or about 3.4. The new CF (3.4) and old CF (3.0) PM_2.5_ values are compared to the regulatory PM_2.5_ values (Table 1).

**Figure 1:**
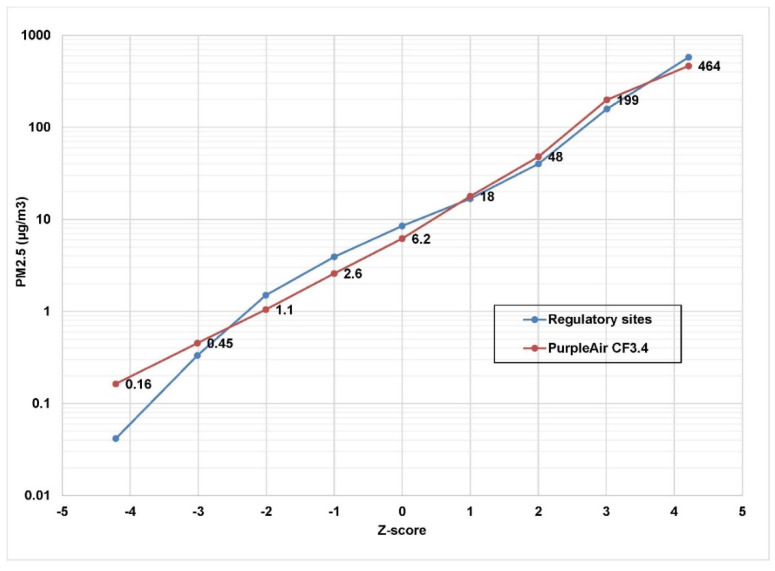
Comparison (using revised CF of 3.4) of 39,474 daily average PM_2.5_ measurements by 182 PurpleAir PA-II outdoor monitors within 0.5 km of 47 Federal and State Agency regulatory monitors in three states. Data labels are shown for the PurpleAir sites. The associated regulatory monitor values are not displayed but can be read from the graph. The x-axis shows the normal probability standard deviations (Z-score). A Z-score of zero corresponds to the median value.

In Figure 1, we have applied an upward correction of 12% (new CF 3.4) to the PurpleAir PA-II outdoor monitors. Figure 1 displays selected percentiles for all 39,474 daily mean outdoor PM_2.5_ measurements from the PurpleAir data using the new CF of 3.4 matched with the AQS data. The data are displayed in terms of standard deviations of the normal probability curve (the Z-score). That is, the median daily average value is plotted at 0, the 16th and 84th percentiles at −1 standard deviation and +1 standard deviation, etc. The maximum outdoor PurpleAir daily average was 464 µg/m3 and the minimum daily average was <0.1 µg/m^3^. Reasonable agreement with the regulatory monitors is evident throughout the entire range of concentrations from <0.1 µg/m^3^ to 464 µg/m^3^. On this log-normal probability graph, a log-normal distribution would appear as a straight line. Although not perfectly straight, there is a fair approximation to a log-normal distribution. A physical explanation of why many environmental datasets display a nearly log-normal distribution has been provided [16].

**Figure 2:**
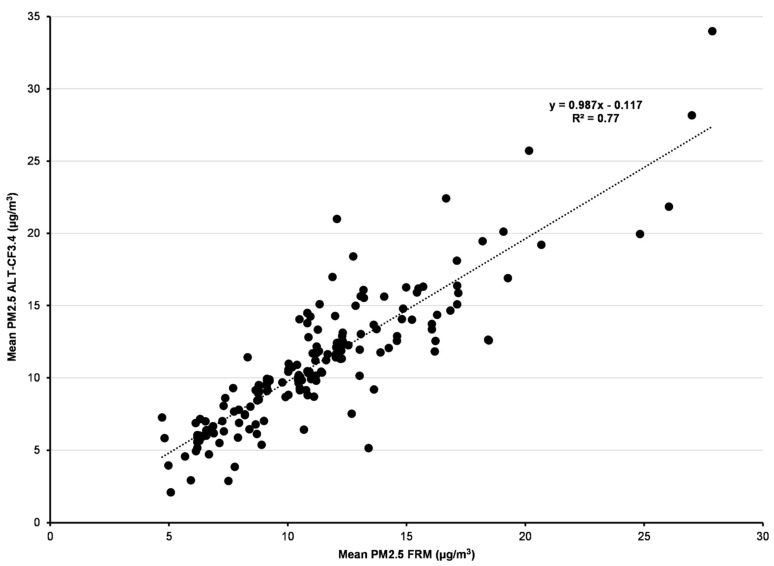
Overall mean PM_2.5_ PurpleAir ALT-CF3.4 vs. mean PM_2.5_ at matched FRM sites within 0.5 km. All 182 matched pairs of sites were required to have ≥30 full days (≥18 h per day).

A plot of PurpleAir monitors with revised CF3.4 vs. FRM PM_2.5_ overall mean values at each pair of sites shows a slope of nearly 0.99 with R^2^ = 77% (Figure 2).

The effect of distance from an FRM monitor was estimated using distances of 1, 2, 5, and 10 km (Figures S3–S7). The R^2^ values were 0.63, 0.63, 0.68, and 0.72, respectively. The slopes were 0.94, 0.87, 0.99, and 1.04, respectively. Spatial variation was quite small, as has been noticed by others [10].

### 3.2. New Calibration of the Outdoor PA-I Monitor with PMS 1003 Sensor vs. Outdoor PA-II Monitors

We located 194 outdoor PA-II sites within 1 km of 43 outdoor PA-I sites (one km was chosen instead of 0.5 km to increase the number of sites available for analysis). A total of 117 pairs of sites, including 26 unique PA-I outdoor sites met our quality assurance requirements. The PA-II monitors were required to have a precision of better than 20% [13,14]. The paired sites produced 24,924 daily average PM_2.5_ concentrations for matched pairs of outdoor PA-I and PA-II monitors with each pair having from 33 to 365 days of valid data. Since the PA-I monitors do not have a second sensor, we minimized possible PA-I outliers by requiring that PA-I values differ by no more than a factor of 3 from the associated PA-II values. This resulted in a final dataset of 23,120 days. The factor of 3 was chosen as the largest factor that retained stability in the mean values; higher values were capable of distorting the distributions. Mean (standard error) values of PM_2.5_ for the 23,120 days were 5.81 (0.07) µg/m^3^ for the PA-I monitors and 6.04 (0.07) µg/m^3^ for the PA-II monitors.

We regressed the daily mean concentrations for PA-I monitor on the PA-II monitor for all 117 joint pairs. All slopes were significantly different from zero. The median (IQR) slope was 0.98 (0.90–0.99) and the median (IQR) R^2^ estimate was 0.96 (0.81–0.98) (Table 2).

**Figure 3:**
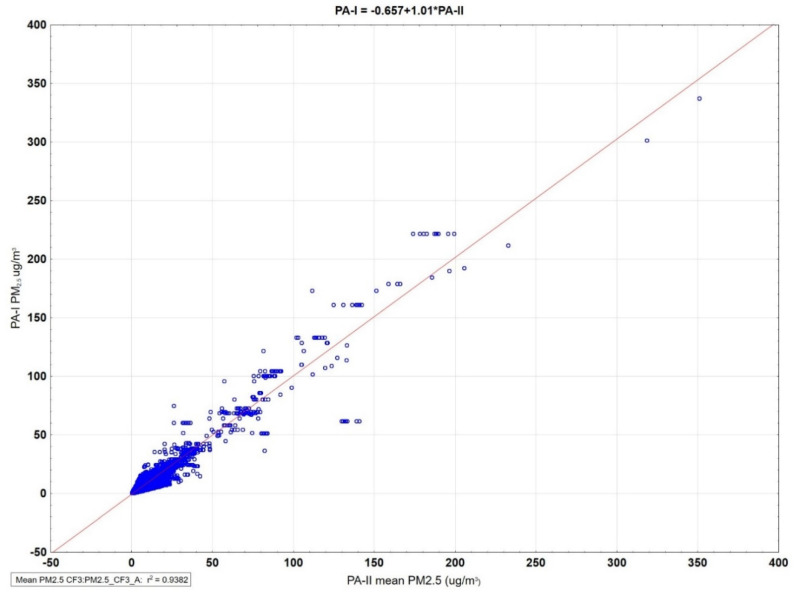
PA-I outdoor overall mean PM_2.5_ averages are regressed against matched mean PA-II outdoor averages at 117 sites less than 0.5 km distance apart. All matched pairs of sites were required to have ≥30 full days (≥18 h per day).

Minimizing either the absolute error or the sum of squared errors resulted in an estimated PA-II/PA-I ratio of 1.04 (MAE = 1.21 µg/m^3^) or 0.96 (RMSE = 2.82 µg/m^3^). That is, the PA-I monitor measurements agreed to within ±4% of the PA-II monitor measurements, and therefore we can assign the same calibration factor of 3.4 to the PA-I monitors. The overall mean PM_2.5_ values for the 117 PA-I monitors are regressed against the overall mean PM_2.5_ values of the matched PA-II monitors (Figure 3). The slope was 1.01 with an R^2^ of 94%.

## 4. Discussion

### 4.1. Comparison with Other Algorithms and Effect on the AQI

Published calibration factors based on the Plantower CF_1 algorithm, such as the value of 0.53 [12] and the EPA value of 0.54 [11], all indicate that the CF_1 algorithm overestimates PM_2.5_ concentrations, perhaps by nearly a factor of 2. Similarly, the ALT-CF3 algorithm, which is not based on CF_1, is 0.55 times CF_1. The EPA Air Quality Index (AQI) is a useful way of gauging outdoor air quality. Community information systems use color shadings to indicate the air quality on a real-time basis. The PurpleAir maps show the Plantower CF_1 PM_2.5_ concentrations with the appropriate color shadings as a default. However, the evidence is that these concentrations overestimate true concentrations and, therefore, people receive air quality estimates that are worse than actual levels. PurpleAir offers other algorithms as alternatives, but default options may be those accepted by most people.

### 4.2. Limitations

All optical monitors will respond to the increase in aerosol diameter due to an increase in RH [17]. The effect is expected to manifest itself strongly for RH in the 60–80% range. However, PurpleAir PA-II monitors have been shown to maintain an internal temperature of approximately 8 °C above ambient temperatures and a corresponding *decrease* of about 15 percentage points in RH [13]. The mean RH of 33 outdoor PA-II monitors in [13] had a diurnal variation of 30–56% RH, never reaching the level at which deliquescence is observed for common atmospheric salts such as KCl, Na_2_SO_4_, and (NH_4_)_2_SO_4_ [18]. Another study observed a rather small effect at RH levels below 50% [19]. For long-term studies such as this one, the variation of RH over the multiday monitoring periods will tend to dilute the effect of high RH for a few hours in a minority of days. Therefore, we have not attempted to incorporate a correction for RH. Another recent study of 1400 PurpleAir monitors in northern and southern California at times of wildfires found the RH effect was too small to include in their model [12].

## 5. Conclusions

About 83 million hourly averages of outdoor PurpleAir PM_2.5_ data in three states over a 4.7-year period were downloaded from the PurpleAir API site. A total of 77 million observations (92.8%) met the requirement of precision to be better than 20%. These hourly values were further averaged to form about 3.5 million daily outdoor averages and compared to about 66,000 daily averages at nearby Federal and State Agency regulatory monitors. The database can be used for further analyses, such as for hourly, seasonal, or geographic variation of PM_2.5_, or whether the CF varies according to PM_2.5_ concentration. It will be particularly useful for studying indoor–outdoor relationships since indoor measurements are missing from many epidemiological studies.

A revised calibration factor (CF) of 3.4 based on comparisons of 182 outdoor PurpleAir PA-II monitors with 47 Federal and State agency regulatory monitors in three states and over the entire 4.7-year time period was developed. The revised CF of 3.4 represents about a 12% increase over the current CF of 3 used in the ALT-CF3 algorithm for PA-II monitors with the PMS 5003 sensor.

A new calibration factor for the PA-I outdoor monitors with a PMS1003 sensor was determined to be within ±4% of the CF for the PA-II outdoor monitors and therefore is also estimated at 3.4. This finding suggests that the >3000 sites with indoor PA-I monitors may now be analyzed in the same way as the nearly 1000 indoor sites using PA-II monitors, since both monitor types have the same calibration factor. This new calibration factor for PA-I monitors effectively quadruples the number of indoor sites available for analysis. Correlations between PurpleAir and FRM monitors were stable across all distances from 1–10 km apart, indicating strong spatial uniformity of PM_2.5_ concentrations.

These results show that PurpleAir data can agree well with regulatory monitors when an optimum calibration factor is applied. 

## Figures and Tables

**Table 1:** Comparison of old and new calibration factors for PA-II monitors with regulatory monitor measurements of PM_2.5_ (µg/m^3^).

	N	Mean	Std. Err.	Min	Z = −1 *	Median	Z = 1 *	Max
PM_2.5_	39,474	11.5	0.076	0.042	3.9	8.5	17	577
PM_2.5_ CF3.4	39,474	11.0	0.090	0.164	2.6	6.2	18	464
PM_2.5_ CF3	39,474	9.9	0.080	0.146	2.3	5.5	16	414

* one normal probability standard deviation below or above the median.

**Table 2:** Statistics for matched outdoor PA-I and PA-II monitors, including calculations of the slopes, R^2^ estimates, standard error of the estimate (µg/m^3^), and the number of days monitored for each pair.

Statistic (N = 117 Sites)	Mean	Std. Err.	Lower Quartile	Median	Upper Quartile	90th Percentile	Max
slope (centered)	0.93	0.0094	0.90	0.98	0.99	0.99	0.999
Adjusted R²	0.87	0.016	0.81	0.96	0.98	0.99	0.998
Std. Err. of Estimate	1.7	0.10	0.81	1.5	2.3	2.8	7.4
Number of days	197	10.4	92	178	303	344	497

## Data Availability

Data are available upon request from the corresponding author at lwallace73@gmail.com.

## Supplementary Materials

The following supporting information can be downloaded at: https://www.mdpi.com/article/10.3390/s22134741/s1, Figure S1: All daily PurpleAir PA-II outdoor PM_2.5_ concentrations (µg/m^3^) between 1/1/17 and 9/8/21 at 9347 sites in three states. Included are 3.5 million daily averages. Figure S2: All FEM/FRM daily outdoor PM_2.5_ concentrations (µg/m^3^) from 1/1/17 through 9/8/21 at 95 sites in three states. N = 66,978 daily averages. Figures S3–S7: Scatterplot regressions of PA-II monitors on FRM monitors at different distances (1, 2, 5, 10 km) apart. Table S1: Outdoor daily PM_2.5_ concentrations (µg/m^3^); Table S2: Days monitored.
