# Supplementary material for: Calibration of PurpleAir PA-I and PA-II Monitors Using Daily Mean PM2.5 Concentrations Measured in California, Washington, and Oregon from 2017 to 2021
Source: Sensors (Basel). 2022 Jun 23;22(13):4741. doi: 10.3390/s22134741 (PMC9269269; doi:10.3390/s22134741)
Supplement: Supplementary file 1 [file sensors-22-04741-s001.zip › sensors-1739887-supplementary.pdf]

Calibration of PurpleAir monitors.

Lance Wallace, Neil Klepeis, and Tongke Zhao

Supplementary Information

Daily outdoor PM2.5 values from 9347 PurpleAir PA-II sites in the states of California, Washington, and Oregon are pictured by date in Figure S1. The time period runs from Jan 1, 2017 to September 8, 2021. Most peak concentrations are due to wildfires.

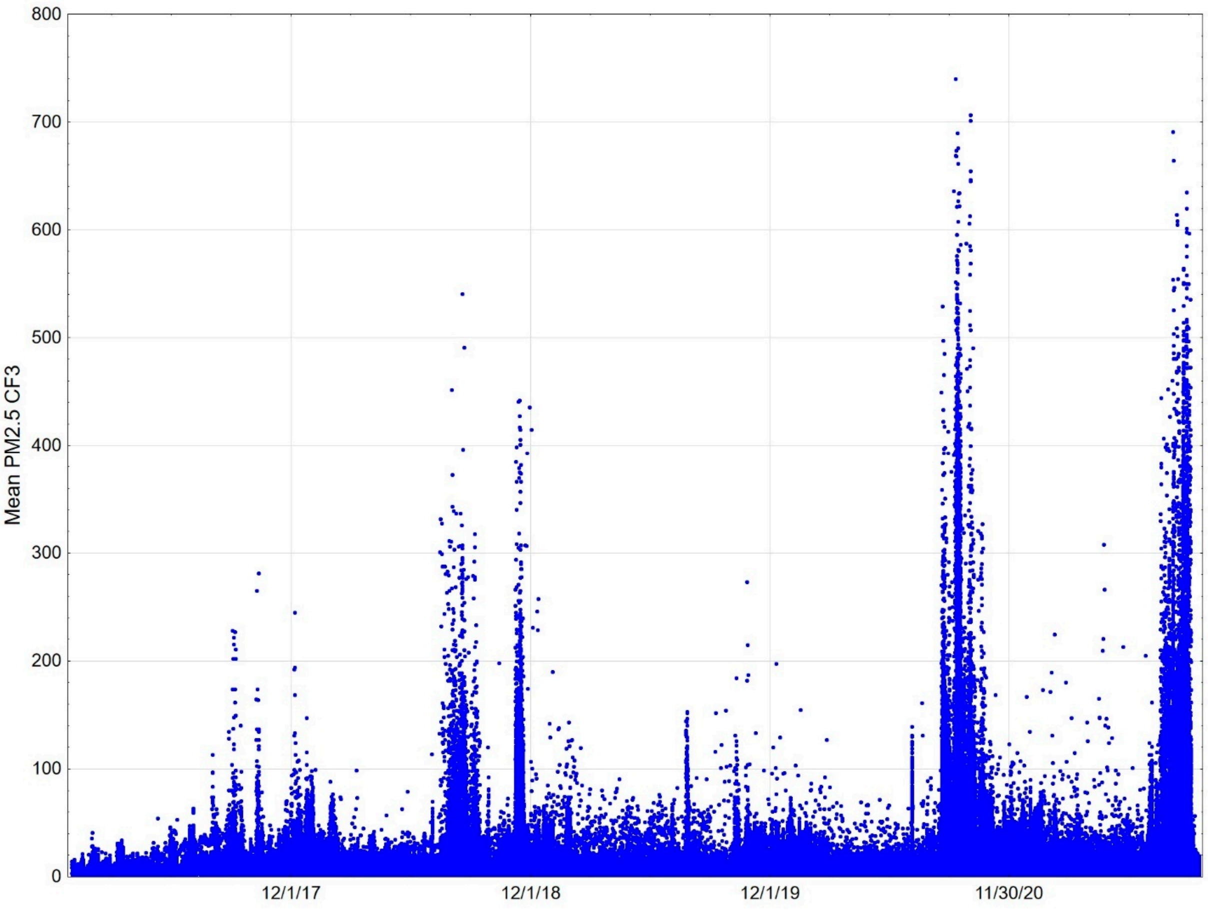

Figure S1. All daily PurpleAir PA-II outdoor PM2.5 concentrations (µg/m^3) between 1/1/17 and 9/8/21 at 9347 sites in three states. Included are 3.5 million daily averages.

Mean daily outdoor $PM_{2.5}$ concentrations as measured by all national and state regulatory sites within 20 km of at least one PurpleAir site are shown by date in Figure S2. The FEM/FRM data consisted of 66,978 daily averaged measurements at 95 sites in the three states.

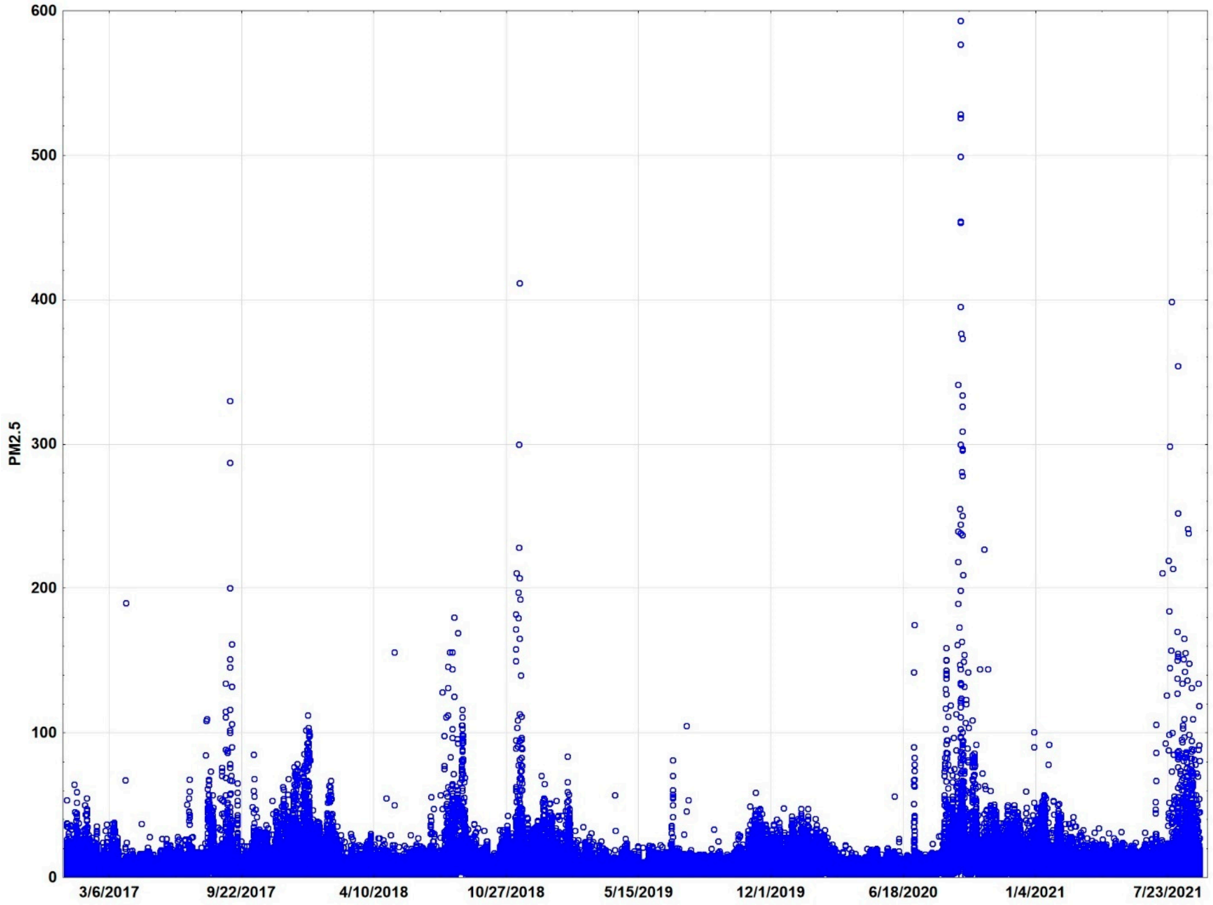

Figure S2. All FEM/FRM daily outdoor $PM_{2.5}$ concentrations (μg/m^3) from 1/1/17 through 9/8/21 at 95 sites in three states. N = 66,978 daily averages.

The daily outdoor mean $PM_{2.5}$ concentrations and related statistics from the PurpleAir and Air Quality System regulatory network in three states over 4.7 years are provided (Table S1). The PurpleAir estimates are based on the ALT-CF3 algorithm (CF=3.0). These are the complete datasets, not matched by geographic proximity, so should not be compared directly.

Table S1. Outdoor Daily $PM_{2.5}$ concentrations (μg/m^3)

	N sites	Mean $PM_{2.5}$	Std. Dev.	10th %tile	Lower quartile	Geom. Mean	Median	Upper quartile	90th %tile	Max
Outdoor PA-II	9347	8.3	4.6	4.5	5.7	7.5	7.4	9.6	12.6	740
FEM/FRM	95	10.0	3.2	6.2	7.9	9.5	9.7	11.9	14.2	590

The median length of time monitored is 305 days for the PurpleAir monitors and 537 days for the regulatory FRM sites (Table S2).

Table S2. Indoor and outdoor days monitored

	N sites	Mean days	Std. Dev.	Min	10th %tile	Lower quartile	Median	Upper quartile	90th %tile	Max
Outdoor PA-II	9347	377	289	30	96	175	305	439	860	1516
FEM/FRM	95	705	469	31	207	380	537	939	1613	1705

104 counties (54 in California, 15 in Oregon, and 35 in Washington) reported at least some daily indoor measurements, although the number of PurpleAir sites was vastly larger in California, resulting in 93% of all daily measurements in the 3 states. Mean values were 4.01 (SE 0.007) ug/m3 in California, 5.15 (0.10) in Oregon and 3.02 (0.03) ug/m3 in Washington. The GM and GSD were 2.3 (2.8), 2.2 (3.0) and 1.5 (3.0) in CA, OR, and WA, respectively.

The "nearest neighbor" computer searches found 313,949 matches of 9347 PA-II outdoor sites within 10 km of the 981 PA-II indoor sites and 1,207,412 matches of the 9347 PA-II outdoor sites within 10 km of 3191 PA-I indoor monitors. There were 2516 matches of 1775 PA-II outdoor sites within 10 km of 95 regulatory (FRM) sites with at least 30 days of joint measurements of daily average $PM_{2.5}$ concentrations.

Effect of distance apart on correlations of PA-II monitors with FRM PM2.5

The following 5 figures (S3-S7) provide x-y plots of PA-II using the revised CF of 3.4 at increasing distances of 1, 2, 5, and 10 km. Correlations (R^2) improved with distance apart from 0.63 to 0.72. However, as the main MS shows, the R^2 for monitor pairs within 0.5 km was higher at 0.77.

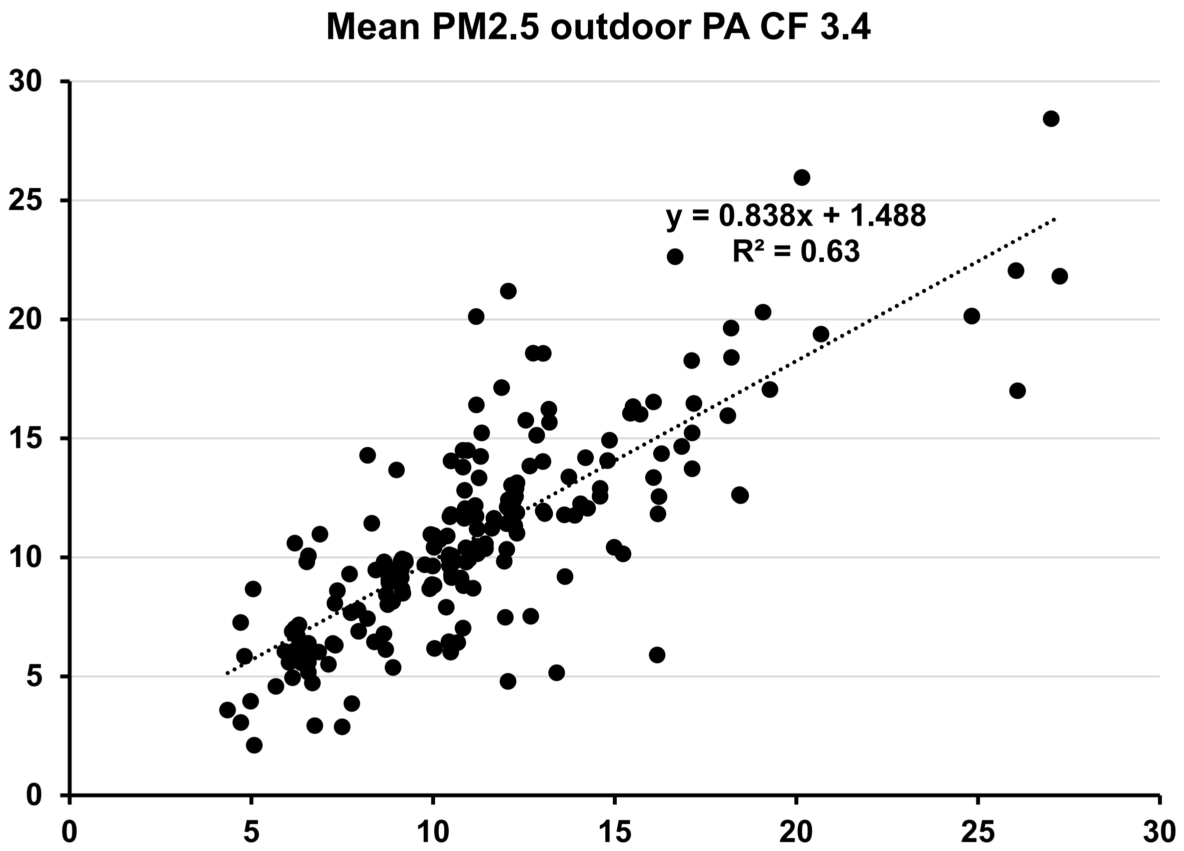

Figure S3. Plot of 206 pairs of PA-II and FRM monitors <1 km apart. For this plot and for succeeding plots through Figure 7, the PA-II is plotted on the y-axis and the FRM on the x-axis. Axis numbers are all in µg/m³.

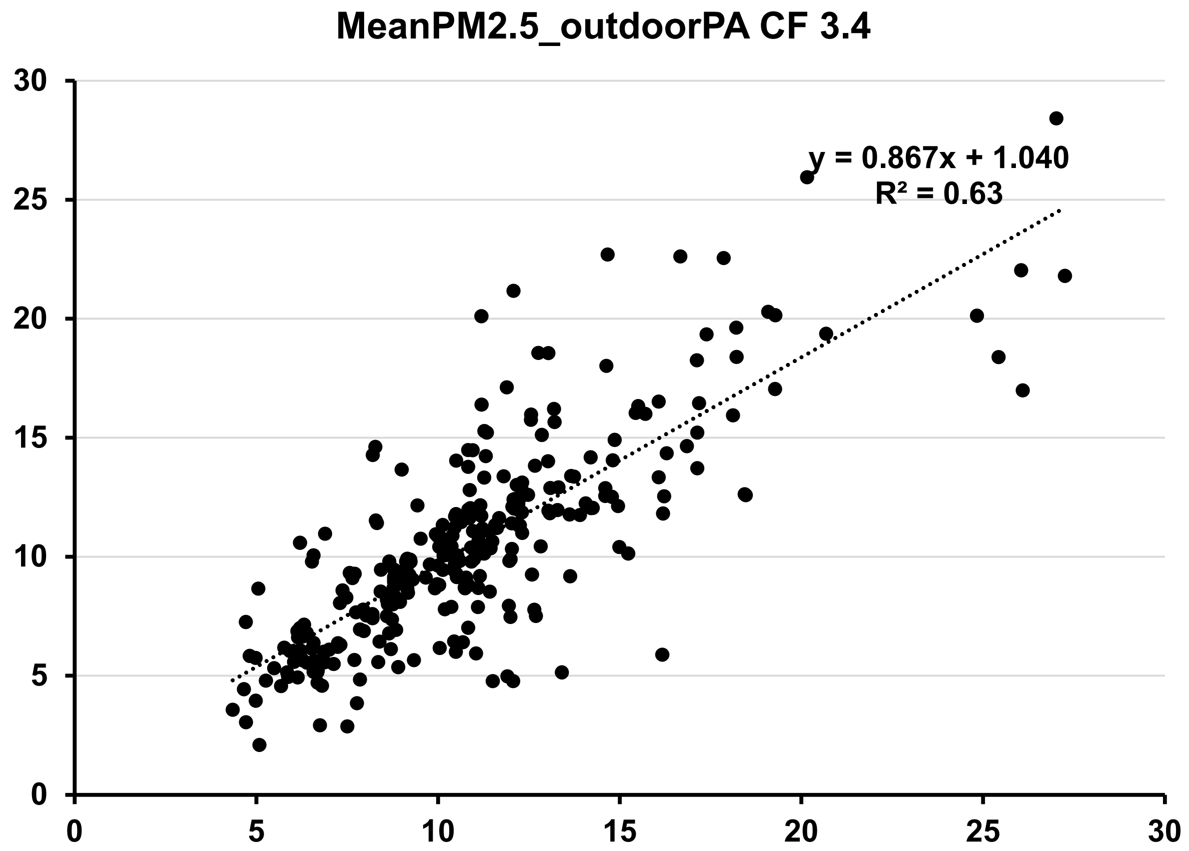

Figure S4. Plot of 299 pairs of PA-II and FRM monitors <2 km apart

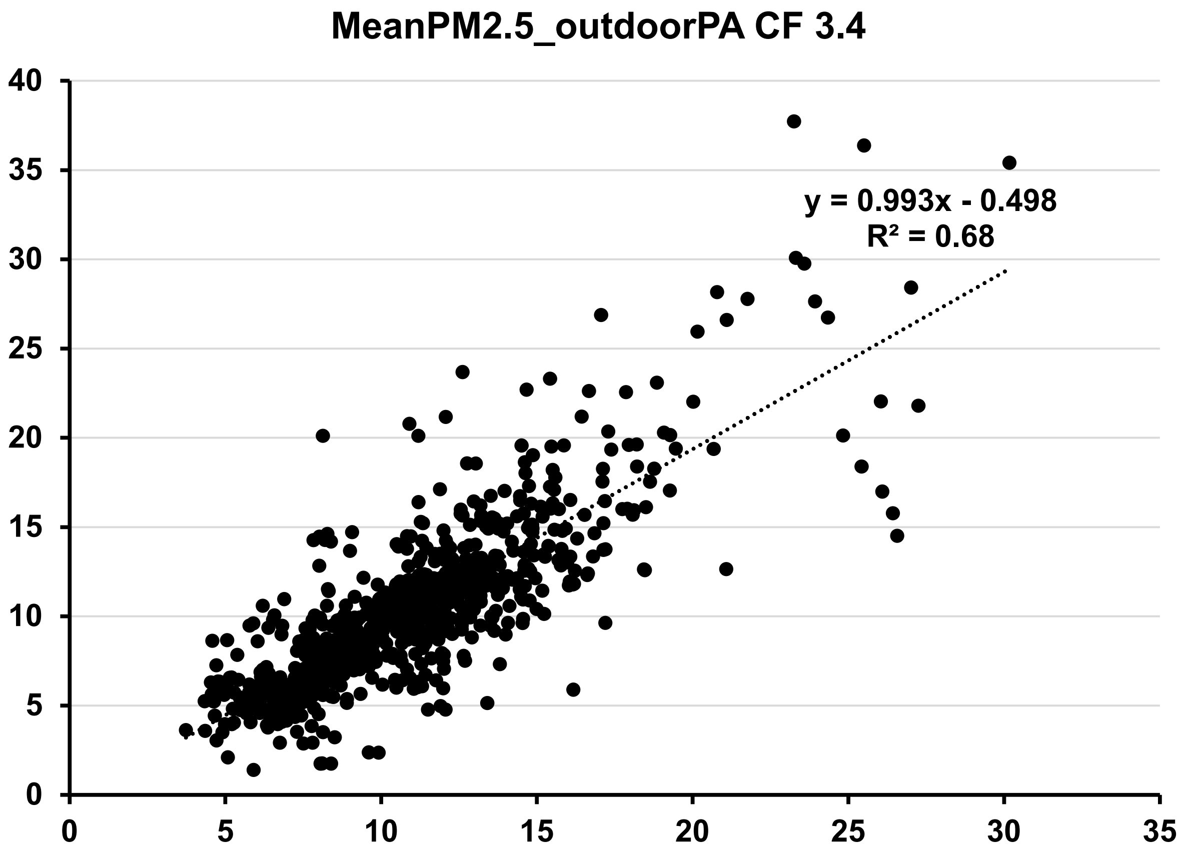

Figure S5. Plot of 920 pairs of PA-II and FRM monitors <5 km apart

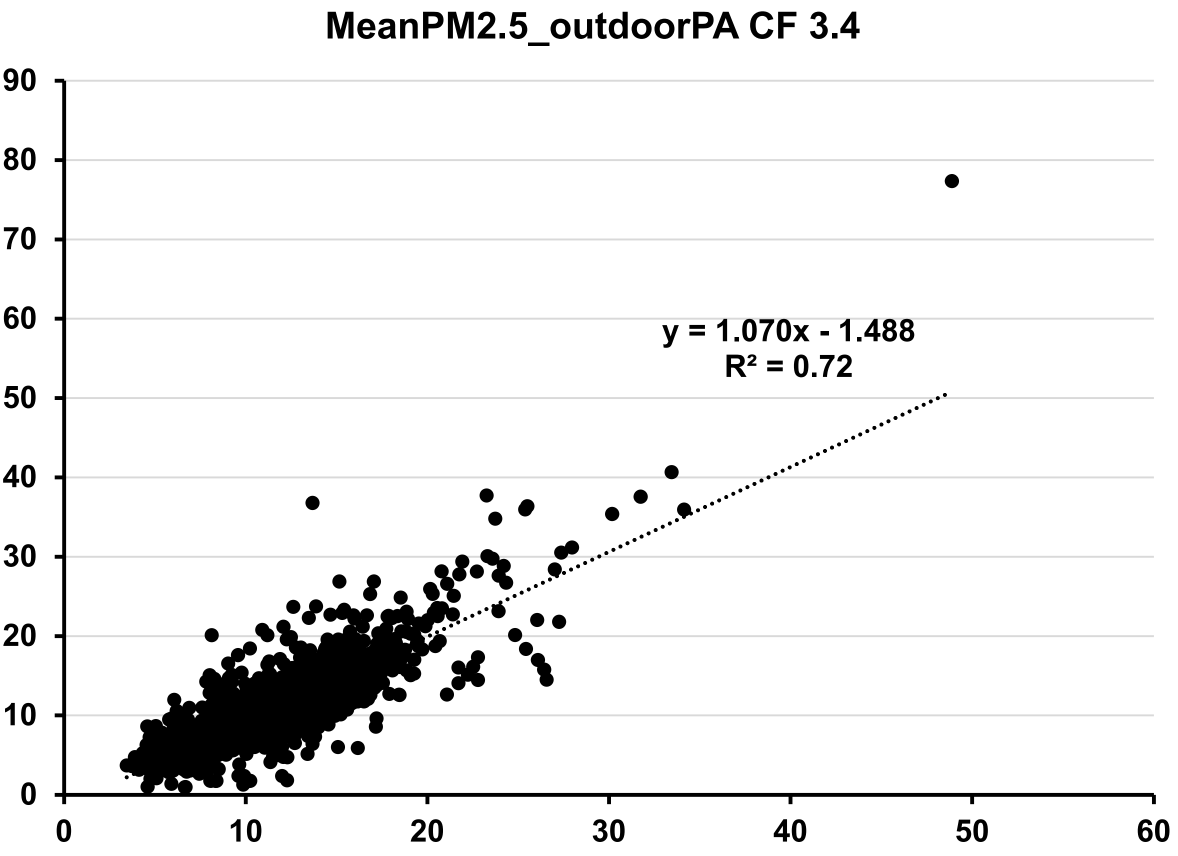

Figure S6. Plot of 2516 pairs of PA-II and FRM monitors <10 km apart

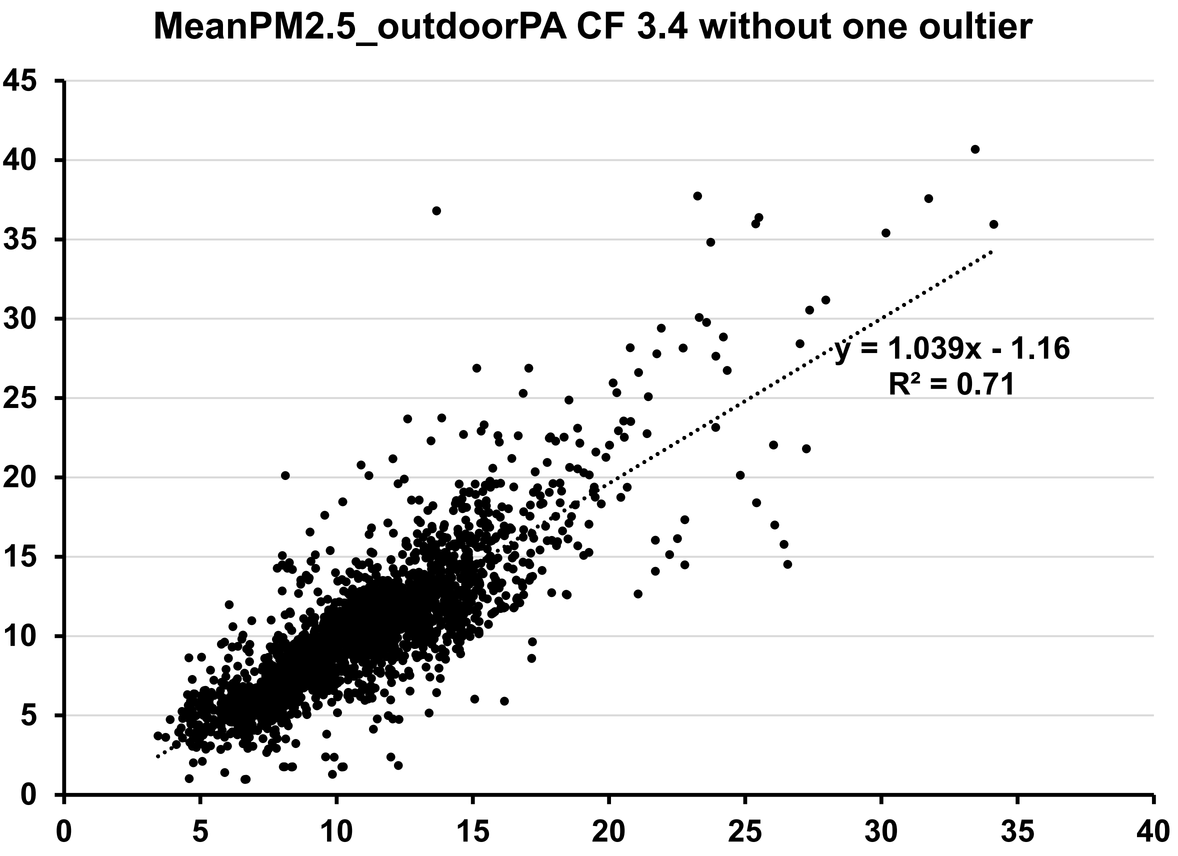

Figure S7. Plot of 2515 pairs of PA-II and FRM monitors <10 km apart omitting one outlier.
